# SOA Amplified 100 Gb/s/λ PAM-4 TDM-PON Supporting PR-30 Power Budget with >18 dB Dynamic Range

**DOI:** 10.3390/mi13030342

**Published:** 2022-02-22

**Authors:** Zhengxuan Li, Yuwen Li, Siyu Luo, Fan Yin, Yuming Wang, Yingxiong Song

**Affiliations:** Key Laboratory of Specialty Fiber Optics and Optical Access Networks, Shanghai Institute for Advanced Communication and Data Science, Shanghai University, Shanghai 200444, China; li_yuwen@shu.edu.cn (Y.L.); siyuluo@shu.edu.cn (S.L.); yinfan970207@shu.edu.cn (F.Y.); wangyuming@shu.edu.cn (Y.W.); herosf@shu.edu.cn (Y.S.)

**Keywords:** optical fiber communication, semiconductor optical amplifier (SOA), passive optical networks (PON), digital signal processing (DSP), pulse amplitude modulation (PAM)

## Abstract

Semiconductor optical amplifier (SOA) is considered an excellent candidate for power amplification at O-band due to its low cost and small footprint. In passive optical networks (PONs), SOA is popular as a booster and pre-amplifier to improve the link power budget. However, whether as a booster or pre-amplifier, SOA will induce different degrees of nonlinearity when the output power is high, which degrades the transmission performance of the system and leads to a limited receiver dynamic range. In this paper, we experimentally demonstrate the feasibility of using SOA in both transmitter and receiver sides for power budget improvement in 100 Gb/s/λ four-level pulsed amplitude modulation (PAM-4) time division multiplexed PON (TDM-PON) system at O-band. For compensating the linear and nonlinear impairments induced by transceivers and SOA, a look-up-table (LUT) pre-compensation at the optical line terminal (OLT) side and a simple feed-forward equalizer (FFE) at the optical network unit (ONU) side are adopted for downstream transmission. For upstream transmission, a 2nd-order Volterra nonlinear equalizer (VNLE) is utilized at the OLT side, and no pre-compensation is used at the transmitter of the ONU, which releases the digital signal processing (DSP) pressure of ONUs in a multi-user scenario. For the soft-decision FEC (SD-FEC) threshold (1 × 10^−2^), the IEEE PR-30 power budget requirement is met, and >18 dB dynamic range is achieved in both 25 km downstream and upstream transmission.

## 1. Introduction

Driven by the rapidly growing demand for emerging applications, such as the internet of things (IoT), 4K/8K-HD video streaming services and virtual reality (VR), the need for higher access network capacity is involved in offering a better user experience. A bunch of broadband fiber access network technologies is evolving to support the accelerating capacity growth requirements. Among these access technologies, the passive optical network (PON) becomes the dominant one due to its cost-effective advantage [1]. In recent years, thanks to the efforts of the PON standardization groups, beyond 10 Gb/s line rate per wavelength of PON systems has successfully been conducted. A system based on 25 Gb/s nominal line rate has been defined by the IEEE 802.3ca 50 G Ethernet Passive Optical Network (EPON) Task Force [2]. The ITU-T Q2/SG15 has also already committed to the standardization of a higher line rate of 50 Gb/s [3]. At C-band, more complex digital signal processing (DSP) is required to achieve high-speed transmission based on direct detection due to high fiber chromatic dispersion (CD) [4]. O-band is a better choice in both high-speed PON downstream and upstream links because its lower CD helps to overcome inter-symbol interference (ISI) [5], and it has been successfully selected as the downstream wavelength of 50G time division multiplexed PON (TDM-PON) [6]. However, O-band suffers higher fiber attenuation loss, which greatly decreases the power budget. Coherent technologies [7] are proposed for improving the loss budget, but for the PON applications, expensive optical components, high DSP complexity and power consumption required by coherent systems make it difficult to be cost-effective. Hard-decision forward error correction (HD-FEC) is one of the simplest and most commonly used FEC technology [8,9]. Recently, more powerful FEC technologies, e.g., soft-decision FEC (SD-FEC), further improved the power budget for high-capacity PON systems [10,11] with an overhead of 15–35% [12].

Semiconductor optical amplifier (SOA) is maturely fabricated and widely used in O-band as a booster and pre-amplifier to improve the power budget [13,14]. The combination of SOA and photodiode (PD) as a receiver provides another solution for high sensitivity detection in PON systems in addition to an avalanche photodiode (APD). Compared with directly modulated laser (DML), electro-absorption modulated laser (EML) has the advantages of lower frequency chirp and slighter CD-induced distortions, which is expected to have a better transmission performance in PON systems. Furthermore, EML and PD are easily integrated with an SOA, which only adds a small cost to the system. SOA will induce the nonlinearity named pattern effect due to gain saturation [15]. For 50 Gbaud four-level pulse amplitude modulation (PAM-4) signals, although the pattern effect is weaker than lower speed signals [16], it still degrades the system performance. Compared with the pattern effect mitigation methods based on optical techniques [17,18,19], schemes using DSP in the electrical domain [16,20] have the advantages of low cost and high flexibility. Look-up-table (LUT) has been proven as a simple and effective algorithm to mitigate the pattern-dependent distortions [21], especially the SOA-induced pattern effect [16]. After optimizing the parameters of LUT, it has good performance and almost no sensitivity penalty. Meanwhile, it is often used as pre-compensation placed in the optical line terminal (OLT) side to reduce the complexity of the optical network unit (ONU) side, which makes it more appropriate for cost-sensitive PON systems. However, most of the current research works only focus on the pattern effect induced by SOA as a pre-amplifier. As a booster that is often used to increase the launch power, SOA will also induce a pattern effect as the output power is high enough. When an SOA booster and an SOA pre-amplifier are used at the same time, the nonlinear impairments will become more serious.

In this paper, we investigate the performance of using SOA as a booster and pre-amplifier for power budget improvement in 100 Gb/s/λ PAM-4 TDM-PON at O-band. In order to mitigate the SOA-induced pattern effect and bandwidth limitation-induced ISI, DSPs are required in both OLT and ONU. However, for cost consideration, most of the complex computations are configured on the OLT side, while only a simple feed-forward equalizer (FFE) is needed for ONU. As a result, an IEEE PR-30 [22] power budget requirement is achieved for both directions, and the dynamic range is as high as 18 dB.

## 2. Principe of SOA-Induced Nonlinearity, FFE-Based LUT and 2nd-Order VNLE

### 2.1. SOA-Induced Nonlinearity

The nonlinear pattern effect induced by SOA comes from the gain saturation, where the SOA gain is defined as the ratio of output power *P_out_* to input power *P_in_*. The amplification principle of SOA is based on stimulated emission. As *P_in_* increases, the carriers in the SOA active region deplete and gain saturation occurs [23]. The SOA gain *G* can be described as:

(1) $$G=G_{0}e^{\frac{P_{out}}{P_{sat}}\left(\frac{G-1}{G}\right)}$$

where $P_{sat}$ is defined as the saturated output power of 3-dB gain compression and $G_{0}$ is the small-signal gain as $P_{in}$ is much smaller than $P_{sat}$. For a small signal, we have $P_{in}\ll P_{sat}$ and $G=G_{0}$ can be derived. When $P_{in}$ increases to exceed $P_{sat}$, the gain is saturated, and the gain saturation induces self-phase modulation (SPM), which is the reason why the pattern effect appears. Therefore, $P_{sat}$ divides the linear and nonlinear regions of SOA.

Because the signal is modulated, the instantaneous input power of SOA changes to time-variant and the instantaneous gain *G* is related to time, which can be written as:

(2) $$G\left(t\right)=\frac{G_{0}}{G_{0}-\left(G_{0}-1\right)e^{-E\left(t\right)/E_{sat}}}$$

with

(3) $$E\left(t\right)=\int_{-\infty }^{t}P_{in}\left(\tau \right)d\tau$$

where E(t) is the fractional energy of an input pulse, and $E_{sat}$ is the pulse energy when SOA is saturated. For the leading-edge of a single pulse whose energy $E_{in}$ is larger than $E_{sat}$, the gain is not saturated and can be expressed as:

(4) $$G_{1}=G\left(-\infty \right)=G_{0}$$

Otherwise, the SOA operates in the nonlinear region for the trailing-edge gain, which can be represented as:

(5) $$G_{2}=G\left(\infty \right)=\frac{G_{0}}{G_{0}-\left(G_{0}-1\right)e^{-E\left(t\right)/E_{sat}}}$$

Therefore, from the perspective of the time-domain signal waveform, the pulse shape is distorted from rectangle to jagged. Additionally, the edge of a pulse affects the former and latter symbols spontaneously and thus, the pulse shape of the current symbol is also related to the several adjacent symbols before and after.

### 2.2. FFE-Based LUT

For each 2*M* + 1 consecutive symbols x(k−M:k:k+M) in the original PAM-*K* signals x(k)∈{0, 1, 2, 3, …, K−1}, it can be mapped to an LUT index and the mapping relationship can be expressed as:

(6) $$i=\sum_{m=-M}^{M}K^{m+M}x\left(k-m\right)$$

2*M* + 1 is defined as the memory length of the LUT. The 2*M* + 1 symbols having the same form compose a pattern, and the same pattern is mapped to the same index. In addition to initializing an LUT of length $K^{2M+1}$ to all zero, another table *C* with the same length as LUT is also needed to count the occurrence number of the same pattern. The error between the middle symbol *x*(*k*) and the corresponding FFE recovered symbol *y*(*k*) is calculated and can be written as:

(7) e(k)=y(k)−x(k)

The error is then accumulated to the index *i* of the LUT, and the index *i* of *C* is increased by 1:

(8) LUT(i)=LUT(i)+e(k)

(9) C(i)=C(i)+1

A sliding window is utilized to slide from the start to the end of *N* consecutive symbols in the transmitted sequence to select 2*M* + 1 consecutive symbols, and then the above steps repeat. *N* is defined as the training sequence length of the LUT. As the sliding window moves to the end, the final LUT can be obtained by the average value of the sum of errors stored in LUT, which is described as:

(10) $$\mathrm{LUT}\left(i\right)=\frac{\mathrm{LUT}\left(i\right)}{C\left(i\right)}$$

**Figure 1:**
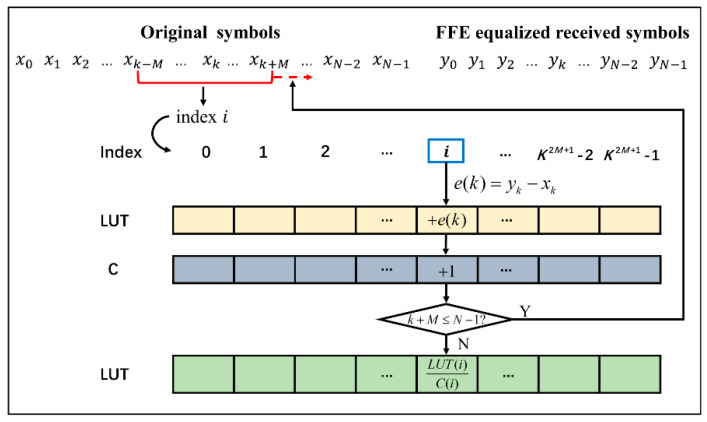
Process of LUT generation.

Figure 1 shows the brief process of training an LUT. The completed LUT is placed at the transmitter to pre-compensate the transmitted signal. Similarly, an LUT index *i* can be mapped from the real original transmitted sequence, and the final transmitted symbol is the difference between the original symbol and the error stored in the index *i* of the LUT, which is:

(11) $$X^{\prime }\left(k\right)=X\left(k\right)-\mathrm{LUT}\left(i\right)$$

### 2.3. 2nd-Order VNLE

The input-output relationship of the 2nd-order Volterra nonlinear equalizer (VNLE) can be written as:

(12) $$y\left(n\right)=\sum_{w=0}^{L_{1}-1}h_{1}\left(w\right)x\left(n-w\right)+\sum_{v=0}^{L_{1}-1}\sum_{w=0}^{L_{2}-1-v}h_{2}\left(w,v\right)x\left(n-w-v\right)$$

where *x*(*n*) and *y*(*n*) denote the input and output of VNLE. *h*_1_ and *h*_2_ represent the first-order and second-order coefficients. *L*_1_ and *L*_2_ are the memory length of the linear and nonlinear parts of the 2nd-order VNLE, respectively.

Since the nonlinearity induced by SOA is complex, the 2nd-order VNLE cannot mitigate it completely. The other important role of the 2nd-order VNLE is to reduce ISI and modulator- and transceiver-related nonlinearities such as square law detection in PD. The result will be presented in detail in Section 4.2.

## 3. Experimental Setup and Offline DSP

### 3.1. Experimental Setup

**Figure 2:**
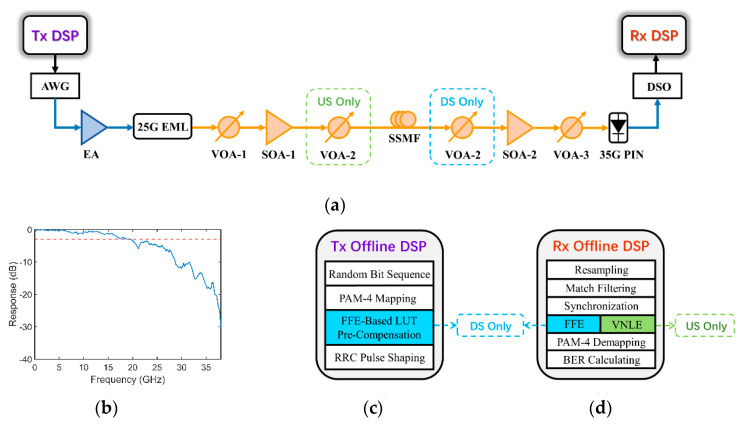
(**a**) Experimental setup for 100 Gb/s/λ PAM-4 PON; Frequency response of the system (**b**); Tx (**c**) and Rx (**d**) side offline DSP flow, respectively.

Figure 2a presents the experimental setup for a 100 Gb/s PAM-4 transmission system based on SOA booster and pre-amplifier at O-band. On the transmitter side (Tx), the 50 Gbaud PAM-4 signal is uploaded into an arbitrary waveform generator (AWG, Keysight M8196A, Santa Rosa, USA) running at 92 GSa/s with 32 GHz 3-dB bandwidth and then amplified by a 55 GHz electrical amplifier (EA) to drive a 25 Gb/s EML operating at a center wavelength 1304.51 nm. The output power of the EML is about 4.3 dBm. To support the 29 dB power budget required in IEEE PR-30, an SOA booster (Aeon SAO11b) working at a bias current of 150 mA is utilized to amplify the launch power, which has a small-signal gain of 14.4 dB and noise figure of 5.9 dB. In order to avoid deep saturation, a variable optical attenuator (VOA) is placed after EML to adjust the input power of the SOA booster to 2 dBm, where a maximal launch power of 12 dBm is obtained. The optical signals are then transmitted over 25 km standard single-mode fiber (SSMF). VOA-2 is placed before and after fiber transmission for upstream and downstream, respectively, to imitate the function of the splitter.

On the receiver side (Rx), a 35 GHz PIN-PD (Newport 1474-A) is applied for optoelectric conversion. To further improve the power budget, an SOA pre-amplifier SOA-2 (Aeon SAO29p) is adopted before direct detection. The VOA-3 is used to attenuate the output of SOA-2 to fix the input power of PIN to 0 dBm. The output signal of PIN is captured by an 80 GSa/s real-time digital storage oscilloscope (DSO) with a 33 GHz bandwidth and processed by offline DSP. The measured frequency response of the whole system is shown in Figure 2b, and the 3-dB bandwidth is about 19 GHz.

### 3.2. Offline DSP

**Figure 3:**
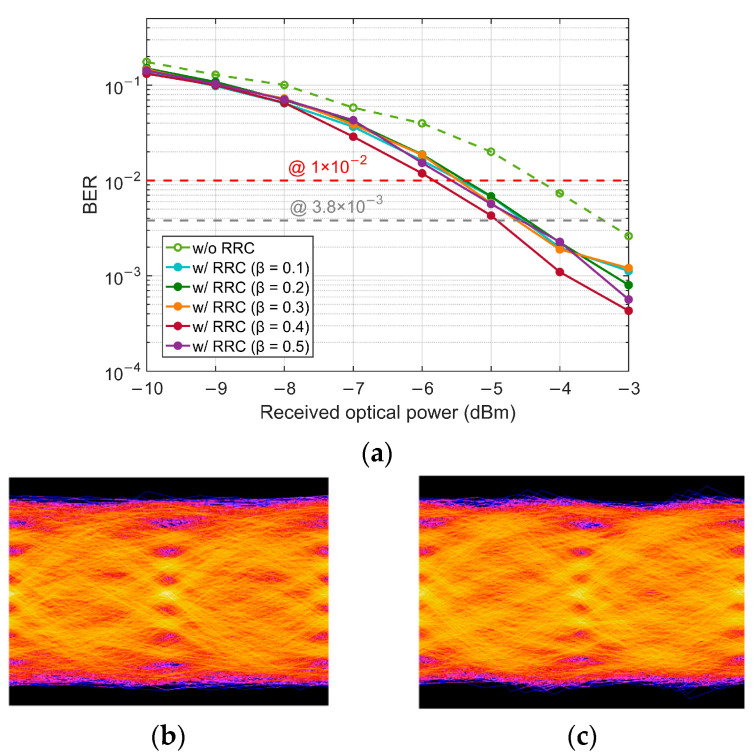
(**a**) BER of different roll-off factors of RRC filter under the case of BtB and without SOA; Eye diagrams of without RRC (**b**), and with RRC (**c**) with a roll-off factor of 0.4.

The offline DSP flow chart at the Tx is shown in Figure 2c. The gray-mapped PAM-4 symbols are obtained from a random bit sequence generated by the Mersenne twister algorithm to get more convincing bit-error-ratio (BER) results [24]. For downstream transmission, the PAM-4 signal is pre-compensated by an FFE-based LUT on the OLT side. While for upstream transmission, no pre-compensation or pre-equalization is used. Since the bandwidth limitation of the transceiver devices can cause ISI, Nyquist pulse shaping based on root-raised-cosine (RRC) filter is adopted with the optimal roll-off factor 0.4. The BER performance for different roll-off factors under the back-to-back (BtB) and without SOA case is shown in Figure 3a. Figure 3b,c show the electrical eye diagrams of 50 Gbaud PAM-4 signal before and after RRC.

The offline DSP flow chart at the Rx is shown in Figure 2d. The captured signal is resampled to 1-sps with the matched filter, and then synchronization is conducted to remove the timing offset and extract the transmitting data for BER calculating. For downstream transmission, only an FFE is applied at the ONU side for decreasing DSP complexity. For upstream transmission, a 2nd-order VNLE is used at the OLT side to reduce linear and nonlinear impairments. The training sequence lengths of FFE and VNLE are both 1500 in our experiments. Finally, all the BER results are calculated based on 100,000 symbols after PAM-4 demodulation.

## 4. Experimental Results and Discussions

**Figure 4:**
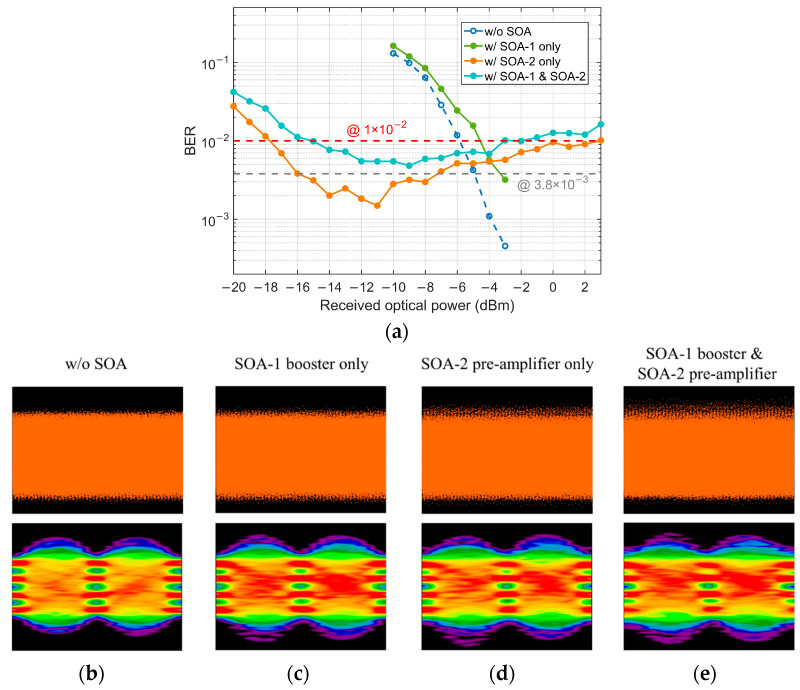
(**a**) BER performance under four conditions: without SOA, with SOA-1 only, with SOA-2 only, and with both SOA-1 and SOA-2; (**b**–**e**) Corresponding receiver time-domain signals and FFE recovered eye diagram at −3 dBm ROP.

The pattern effect appears as the ‘eye’ closure of the higher levels on an eye diagram for PAM signals. Firstly, to investigate the influence of pattern effect induced by SOA-1 and SOA-2 on the 50 Gbaud PAM-4 signal, we test the BER performance with FFE under four BtB conditions—without SOA, with SOA-1 only, with SOA-2 only, with both SOA-1 and SOA-2, and the result is presented in Figure 4a. Figure 4b–e show the received time-domain signals and corresponding FFE recovered eye diagrams at a received optical power (ROP) of −3 dBm. It can be seen from the eye diagrams that both SOA-1 and SOA-2 introduce distortions, and the influence of SOA-2 is more serious than SOA-1. The BER curves also prove that when two SOAs are used, the signal suffers more distortions compared with the cases using one SOA. As a booster, the current adjustment of SOA-1 has little effect on the results, so we only focus on the performance optimization of SOA-2 in the following experiments.

**Figure 5:**
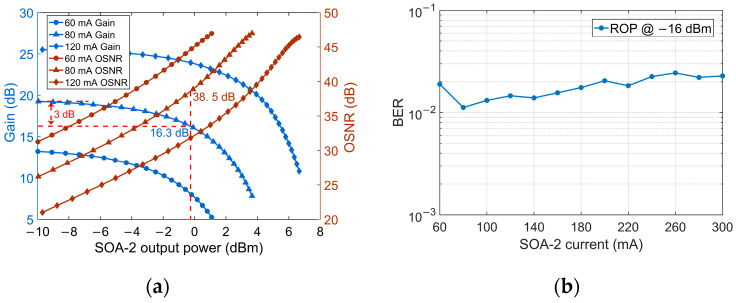
(**a**) SOA-2 gain and OSNR of 100 Gb/s PAM-4 signal versus output power; (**b**) BER of 50 Gbaud PAM-4 signal versus SOA-2 bias current.

For optimizing the performance of SOA-2 as a pre-amplifier, the SOA gain and optical signal-to-noise ratio (OSNR) of 50 Gbaud PAM-4 signal versus optical output power with different bias currents are measured and shown in Figure 5a. For the 60 mA bias current, despite the high OSNR, the SOA gain is not high enough to support a high input power of PIN when the ROP is low, which will result in poor BER performance. Considering higher bias current induces higher amplified spontaneous emission (ASE) noise [25], the SOA-2 bias current is finally optimized to 80 mA with −0.2 dBm $P_{sat}$ and corresponding −16.5 dBm saturated input power to strike a balance between the gain and OSNR. Figure 5b shows the BER versus SOA-2 bias current under the BtB test at −16 dBm ROP. Varying the bias current from 80 mA to 300 mA, the BER shows an increasing trend.

### 4.1. Downstream Transmission

**Figure 6:**
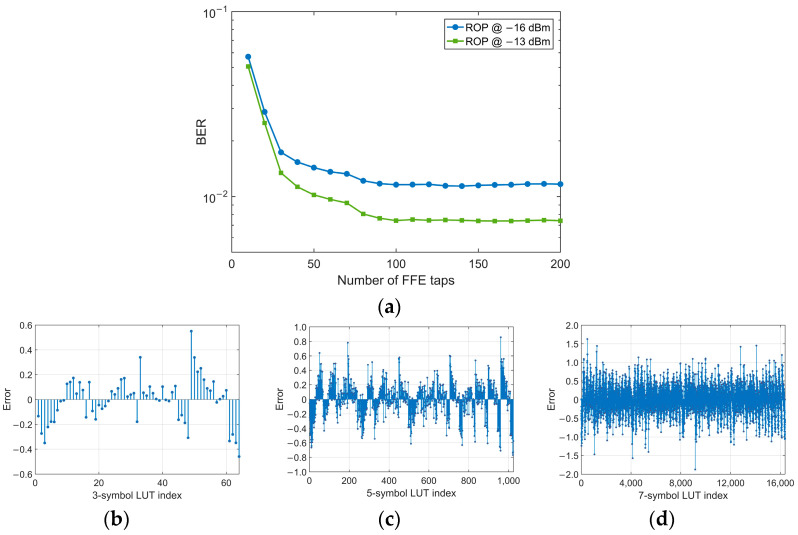
(**a**) BER performance as a function of the number of FFE taps; LUTs with memory length 2*M +* 1 = 3, 5 and 7 for (**b**–**d**), respectively.

For the downstream link, we firstly investigate the required number of FFE taps at the Rx side. As shown in Figure 6a, the BER performance is improved as the number of taps is increased but stabilizes when the number exceeds 100. So we fix the number of FFE taps to 100 in the following experiments. Afterward, under the condition that the training sequence length of LUT is 100,000, we use this FFE to generate three LUTs with different memory lengths—3, 5 and 7, respectively, and the generated LUTs are presented in Figure 6b–d.

**Figure 7:**
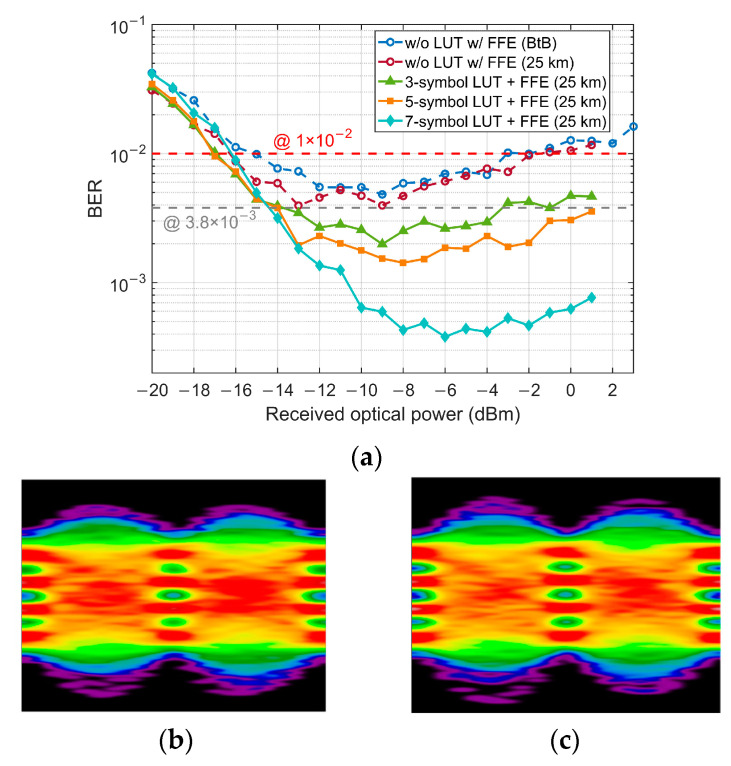
(**a**) BER of post FFE combined with several LUTs. Eye diagrams with 3- (**b**) and 5-symbol (**c**) LUT pre-compensation and FFE at −3 dBm ROP.

Figure 7a depicts the BER performance with FFE-based LUT pre-compensation. Since the O-band has a very low CD coefficient, the system performance is hardly degraded by CD in a short distance (e.g., 25 km) SSMF transmission. Besides, for a short distance and low launch power, the fiber nonlinearities can also be neglected. It is worth noting that contribution to the interaction between CD and chirp effects of the modulator. The BER performance is slightly improved after 25 km transmission compared with the BtB case. To better mitigate the pattern effect, the calibration ROP point is chosen to −8 dBm where the pattern effect is strong, and the BER is lower than the SD-FEC threshold (1 × 10^−2^). As the results shown in Figure 7a demonstrate, thanks to larger memory length, the 7-symbol LUT has a much stronger performance to mitigate the pattern effect than 3- and 5-symbol LUT. However, owing to the large error value stored in 7-symbol LUT, it has a potential risk of sensitivity penalty. Moreover, its higher calculation complexity and huge memory requirement make it difficult to be implemented practically. Notice that for 3- and 5-symbol LUT combined with FFE, a 0.8 dB receiver sensitivity improvement is obtained compared with FFE only and the combination of 7-symbol LUT and FFE. Therefore, a 3 or 5-symbol LUT will be a better choice to trade off the performance and complexity. However, for the SD-FEC threshold, 3- and 5-symbol LUTs have the same performance of sensitivity and dynamic range, so 3-symbol is finally chosen in our experiment. Figure 7b,c show the eye diagrams after 25 km SSMF transmission with 3- and 5-symbol LUT pre-compensation and post FFE, respectively, and the ‘eye’ between levels ‘2’ and ‘3’ is clearly open.

### 4.2. Upstream Transmission

**Figure 8:**
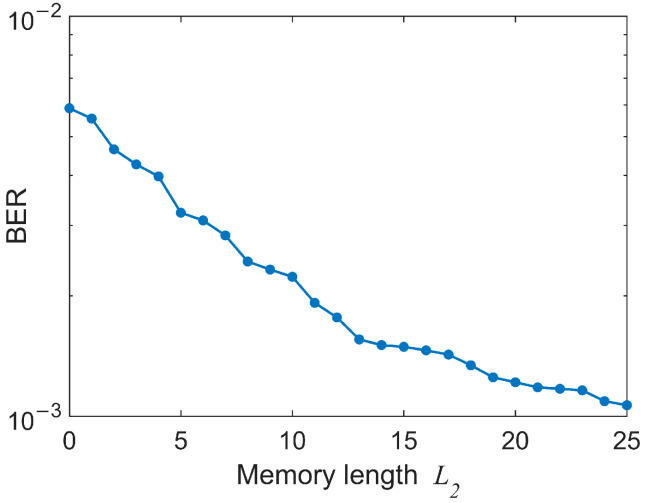
BER versus the memory length *L*_2_ of VNLE with *L*_1_ = 100 at −8 dBm ROP.

**Figure 9:**
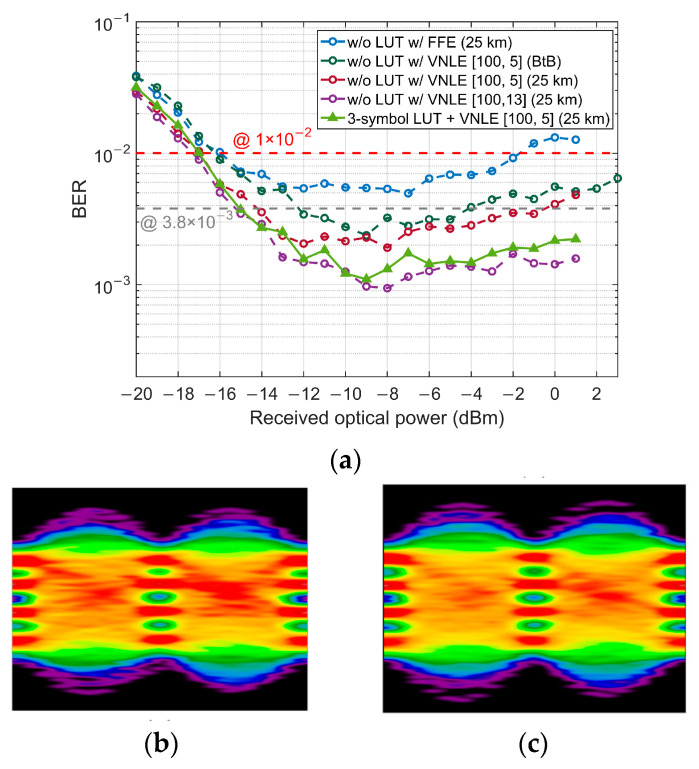
(**a**) BER performance of VNLE only and LUT combined with VNLE; Eye diagrams with VNLE (*L*_2_ = 5) only (**b**) and with 3-symbol LUT and VNLE (*L*_2_ = 5) (**c**) at −3 dBm ROP.

For upstream direction, considering the memory requirement and computational complexity, we did not apply LUT at the transmitter of the ONU. In this case, simple linear FFE at the Rx will result in a limited dynamic range. Therefore, a nonlinear VNLE is utilized at the receiver of the OLT instead. For the 2nd-order VNLE, we continue to use the optimized FFE as its linear part. Similarly, we measure the BER performance versus the memory length *L*_2_ of VNLE with *L*_1_ fixed to 100 at −8 dBm ROP. As depicted in Figure 8, the BER decreases as we increase *L*_2_. Yet, good receiver sensitivity and dynamic range can be achieved when *L*_2_ = 5 in consideration of the SD-FEC threshold. Figure 9a shows the results of *L*_2_ = 5 and *L*_2_ = 13 for comparison. So *L*_2_ is finally set to 5.

As shown in Figure 9a, with the assistance of VNLE, all BERs are below the SD-FEC threshold when ROP is greater than −17 dBm. However, the eye diagram in Figure 9b shows that the pattern effect has not been completely eliminated. By combining LUT with VNLE, the pattern effect can be better mitigated, as Figure 9c shows. However, it brings no benefit to the sensitivity and dynamic range under the SD-FEC threshold. So, as long as VNLE is used at the OLT Rx, LUT becomes unnecessary in the ONU Tx.

Table 1 is a summary of some key parameters in the system. Table 2 and Table 3 summarize the receiver sensitivity, power budget, dynamic range and computational complexity of 25 km downstream and upstream transmission with different equalization algorithms, respectively. Considering the SD-FEC threshold, a receiver sensitivity of −17 dB and a dynamic range of >18 dB are both achieved. The achieved 29 dB power budget meets the PR-30 requirement.

## 5. Conclusions

We have demonstrated 100 Gb/s/λ PAM-4 TDM-PON downstream and upstream transmission at O-band based on EML + SOA transmitter and SOA + PIN receiver, and PR-30 power budget requirement is reached after 25 km fiber transmission. To reduce the complexity of ONUs, different DSP algorithms are utilized for downstream and upstream transmission. For downstream transmission, compared with only ONU-side 100-tap FFE, the introduced 3-symbol LUT pre-compensation at the OLT side effectively reduces the pattern effect induced by SOA booster and pre-amplifier, which contributes a 0.8 dB receiver sensitivity improvement and >3 dB dynamic range extension. For upstream transmission, without any ONU-side pre-compensation and pre-equalization, >3 dB dynamic range is improved with the help of the 2nd-order nonlinear part of the OLT-side VNLE by contrast with linear FFE. The power budget can be further improved by using an SOA with higher saturation power as a booster or by applying DSP with higher complexity at the transceiver sides.

## Figures and Tables

**Table 1:** Key parameters in the system.

Direction	Roll-Off Factor	Launch Power (dBm)	SOA-1 Current (mA)	SOA-2 Current (mA)	Equalization
Downstream	0.4	12	150	80	3-symbol LUT + 100-tap FFE
Upstream					2nd-order VNLE (*L*_1_ = 100 and *L*_2_ = 5)

**Table 2:** 25 km downstream transmission with 3-symbol LUT and 100-tap FFE.

FEC Threshold	Sensitivity (dBm)	Power Budge (dB)	Dynamic Range (dB)	Equalization Complexity (Number of Multiplications)
HD-FEC (3.8 × 10^−3^)	−14	26	10.8	3 + 100 = 103
SD-FEC (1 × 10^−2^)	−17	29	>18	

**Table 3:** 25 km upstream transmission with 2nd-order VNLE (*L*_1_ = 100 and *L*_2_ = 5).

FEC Threshold	Sensitivity (dBm)	Power Budge (dB)	Dynamic Range (dB)	Equalization Complexity (Number of Multiplications)
HD-FEC (3.8 × 10^−3^)	−14.2	26.2	13.7	100 + (5 × 6) = 130
SD-FEC (1 × 10^−2^)	−17	29	>18	
